# Triage of patients with AUS/FLUS on thyroid cytopathology: effectiveness of the multimodal diagnostic techniques

**DOI:** 10.1002/cam4.636

**Published:** 2016-01-18

**Authors:** Tae Hyuk Kim, Dae Joon Jeong, Soo Yeon Hahn, Jung Hee Shin, Young Lyun Oh, Chang‐Seok Ki, Jong‐Won Kim, Ju Young Jang, Yoon Young Cho, Jae Hoon Chung, Sun Wook Kim

**Affiliations:** ^1^ Department of MedicineSamsung Medical CenterSungkyunkwan University School of MedicineSeoulKorea; ^2^ Department of RadiologySamsung Medical CenterSungkyunkwan University School of MedicineSeoulKorea; ^3^ Department of PathologySamsung Medical CenterSungkyunkwan University School of MedicineSeoulKorea; ^4^ Department of Laboratory Medicine and GeneticsSamsung Medical CenterSungkyunkwan University School of MedicineSeoulKorea

**Keywords:** Atypia of undetermined significance, BRAF, cytopathology, follicular lesion of undetermined significance, thyroid nodule

## Abstract

The management of patients with thyroid cytopathologic diagnosis of atypia (or follicular lesion) of undetermined significance (AUS/FLUS) is a complex clinical problem. The purpose of this study was to develop a practical triage scheme based on multiple diagnostic tests in general use. We performed a retrospective cohort study involving 15,335 consecutive patients with a referral diagnosis of thyroid nodule between April 2011 and March 2015 using an institutional database. We obtained 904 patients with an initial cytopathologic diagnosis of AUS/FLUS who underwent repeat fine‐needle aspiration or core needle biopsy, 388 of whom had a corresponding histopathological diagnosis for excised index lesions. The diagnostic performance of ultrasound (US) findings, repeat biopsy, and *BRAF*^V^ ^600E^ mutation in cytopathologic specimens were evaluated individually or as a set. Of the 388 resected AUS/FLUS cases, 338 (87.1%) were thyroid cancer. The positive likelihood ratios (LRs) for *BRAF*^V^ ^600E^ mutation and repeat biopsy result of suspicious for malignant cell (SMC) or worse were 11.6 (95% CI = 1.7–77.8) and 13.7 (95% CI = 4.6–41.0), respectively. The absence of suspicious findings on US combined with cytologic result of less than SMC or negative *BRAF*^V^ ^600E^ mutation produced negative LRs ranging from 0.06 to 0.15, corresponding to negative predictive values of over 90% in both primary and referral settings. For patients with AUS/FLUS cytopathology, clinical decision making can be guided by a simple triage scheme based on US findings, repeat biopsy, or *BRAF*^V^ ^600E^ mutation.

## Introduction

The discovery of thyroid nodules has become a frequent event in clinical practice, as the prevalence of a palpable nodule in the general adult population ranges from 4% to 7%, and the incidence rises to 60% if using a high‐resolution ultrasound (US) machine 1, 2, 3. Because the vast majority of thyroid nodules are benign and can be managed conservatively, it is important to identify suspicious nodules that require surgical excision. Fine‐needle aspiration (FNA) biopsy has been used for this purpose as a reliable, safe, and cost‐effective screening test as its wide implementation in the 1980s. The reporting classification of thyroid cytopathology has evolved and been refined, and the current standard is known as the Bethesda system 4, 5.

However, the management of an equivocal cytopathology test remains a complex clinical and public health problem. The most controversial abnormal biopsy result is one of uncertainty: atypia (or follicular lesion) of undetermined significance (AUS/FLUS) 1, 6. This category was first proposed by the expert committee of the National Cancer Institution Thyroid FNA State of the Science Conference held in Bethesda in October 2007 to represent a group at low risk for malignancy in whom a repeat FNA would be recommended 4. In reality, the AUS/FLUS lies in a gray zone of cytopathology that encompasses atypia due to sample preparation artifact and architectural or nuclear atypia that are not readily assigned to other diagnostic categories. A comprehensive study showed that the reproducibility of AUS/FLUS is only 50%, even among expert thyroid cytopathologists 7. Studies using the current Bethesda system reported a marked variability in the incidence of (0.7–18%) and malignancy rate (6–81%) in resection specimens of AUS/FLUS cases 8, 9.

Given this heterogeneity of malignant risk in the AUS/FLUS category, it is not surprising that current guidelines now allow virtually all possible diagnostic options including observation, repeat FNA, molecular diagnostics beyond the B‐type Raf proto‐oncogene (*BRAF*) V600E mutation, and consideration for diagnostic surgery for definite diagnosis and treatments based on the level of clinical suspicion 10, 11. Therefore, there is an unmet need for an effective triage strategy that is easily applicable in both primary care and the referral setting and will give sufficient information to classify which patients with AUS/FLUS are at risk and require surgery and which patients can be spared the anxiety and costs of intensified diagnostic work‐up.

In this study, we evaluate the utility of US findings, *BRAF* ^V600E^ mutation in a preoperative biopsy sample, and repeat biopsy results individually or as a set to develop a practical triage scheme for patients with an initial cytopathologic diagnosis of AUS/FLUS.

## Materials and Methods

### Study population and cytopathologic interpretation

This study was conducted in a hospital‐based tertiary referral center. The study protocol was approved by the institutional review boards. Beginning in April 2011, our pathology department decided to adopt the six tier categories of the Bethesda System for Reporting Thyroid Cytopathology (BSRTC) for classifying thyroid nodules: 5 nondiagnostic, benign, AUS/FLUS, follicular neoplasm/suspicious for follicular neoplasm (FN/SFN), suspicious for malignant cell (SMC), and malignant. From April 2011 to March 2015, 15,335 patients visited the thyroid clinic with a referral diagnosis of thyroid nodule and underwent US‐guided FNA. Local cytopathologic specimens were collected from 1862 (12.1%) patients. An expert thyroid cytopathologist (O. Y. L.) reviewed equivocal cases and reclassified each nodule according to the BSRTC. The criteria for adequate smear were the presence of six groups of cells with more than 10 cells per group 5. Of the 1567 (10.2%) consecutive patients with an initial cytopathologic diagnosis of AUS/FLUS, 904 (57.7%) underwent repeat biopsy with FNA (786/904, 86.9%) or core needle biopsy (CNB, 152/904, 16.8%) for further characterization of the index nodule as recommended 5. The findings of CNB were grouped into the same categories of BSRTC as previously described 12, 13, 14. For 34 patients with both repeat FNA and CNB results available, a more severe cytopathologic diagnosis was recorded.

Thyroid surgery was performed based on the clinical judgment of the treating physician and their shared decision with patients. A standard histopathological diagnosis was obtained for 388 (42.9%) of these patients. The biopsied nodules were matched to resected nodules according to size and location. Incidental microcarcinomas that did not match were not considered malignant on resection.

### US‐guided FNA and CNB procedures

All FNA or CNB was performed under US guidance by one of seven board‐certified radiologists. The US scanners (IU22; Philips Medical Systems, Bothell, WA) were 5–12 MHz linear array transducers. The detailed procedures of FNA or CNB have been described elsewhere 12. Briefly, the FNA specimens were obtained from the nodule in two or three passes with a 23‐gage needle attached to a 2‐mL syringe with direct US visualization. Aspirated materials were smeared onto frosted‐end glass slides and immediately fixed in 95% alcohol for both Papanicolaou and May‐Grunwald‐Giemsa staining. The CNB specimens were obtained using 1.1 cm excursion, 18 gage, double action spring‐activated, Trucut‐type needle (Acecut; TSK Laboratory, Tochigi‐ken, Japan). The core needle was advanced from the isthmus of the thyroid toward the solid portion of the nodule. After the tip of the needle had been advanced into the edge of the nodule, the stylet and cutting cannula were sequentially fired and at least two or three biopsy cores were obtained. The biopsy specimens were fixed in formalin solution. No major complications were noted in any patients receiving FNA or CNB procedures.

### Analysis of US findings

The morphology, location, and maximal size of the nodule were reported for each thyroid nodule. All US images were retrospectively reviewed by two board‐certified radiologists (S. J. H. or H. S. Y.) until consensus was reached. The US findings were categorized into two groups for convenience: US‐suspicious and US‐nonsuspicious. Thyroid nodules with one or more of the following high‐risk features were considered US‐suspicious: irregular (infiltrative) margins, microcalcifications, taller than wide shape, marked hypoechogenicity less than the surrounding strap muscle, and disrupted rim calcifications with extrusive soft tissue component 15, 16, 17.

### Detection of *BRAF* ^V600E^ mutation in biopsy samples

The decisions to perform *BRAF* ^V600E^ mutation as an ancillary test in the residual biopsy specimen were at the discretion of both the managing physician and radiologist without specified protocol. The preoperative test results of the index nodule were collected from 44.8% of patients who had histopathologic diagnosis. The malignancy rates between patients with and without test results were similar (90.2% and 84.6%). An early seminal study demonstrated the diagnostic utility of the *BRAF* ^V600E^ mutation in FNA specimens 18, and we then reported clinical implications of highly sensitive detection methods 19. The *BRAF* ^V600E^ mutation was detected using both allele‐specific polymerase chain reaction (AS‐PCR) and mutant enrichment with 3′‐modified oligonucleotide (MEMO) sequencing, which had a very high sensitivity (detection limit of 0.1%). The results were considered positive if the mutation was detected by either method. Briefly, genomic DNA was extracted from the remaining sample of FNA or CNB after cytopathologic evaluation using a QIAamp DNA minikit (QIAGEN, Chatsworth, Canada). AS‐PCR was performed using either a Seeplex BRAF ACE detection system (Seegene, Seoul, Korea) 19 or a Real‐Q BRAF V600E Detection assay as previously described 20. MEMO sequencing uses a blocking primer, which matches the wild‐type DNA, so the extension of the normal allele does not occur. The primers and PCR conditions of MEMO‐PCR were described elsewhere 21.

### Statistical analysis

For each cytopathologic category in repeat biopsy, we calculated the malignancy rates by dividing the number of confirmed malignancies on excision by the total number of enrolled cases with the assumption that nonexcised nodules were benign. The rates according to the number of patients who underwent excision were also reported. We used histopathological diagnosis after surgery as the gold‐standard reference test and assessed the diagnostic accuracy of the dichotomized tests individually or as a set. Sensitivity, specificity, and positive and negative likelihood ratios (LRs) were calculated using established methods 22, 23. Generally, tests with high‐positive LR (ideally, >10.0) are statistically adequate to find malignant cases, and tests with low negative LR (<0.10) can exclude malignancy 23. Because predictive values are directly influenced by the prevalence of malignancy in nodules with AUS/FLUS cytopathology, which is known to vary widely between medical settings 24, 25, 26, we calculated the positive predictive value (PPV) and negative predictive value (NPV) according to three conceptual levels of pretest probability: (1) 10% (community health center), (2) 25% (secondary to tertiary referral center), and (3) 40% (tertiary referral center). Statistical analysis was performed with the use of MedCalc statistical software version 15.6.1 (MedCalc Software, Mariakerke, Belgium). Confidence intervals for proportions are reported as two‐sided exact binomial 95% confidence intervals. Categorical variables were analyzed by Fisher's exact test. Continuous variables were analyzed by Student's *t*‐test. *P*‐values of <0.05 were considered statistically significant.

## Results

We enrolled 904 consecutive patients who underwent repeat biopsy of a nodule with AUS/FLUS at the initial FNA test. The baseline characteristics of the patients and nodules are shown in Table 1. Patients who underwent surgery for AUS/FLUS nodules were younger, more likely to be referred from community centers, and to have had a repeat biopsy by CNB compared to patients who did not (all *P *<* *0.05). Sex and nodule size did not differ significantly between the total population and the primary study population of the surgical sample (*n* = 388).

**Table 1:** Baseline demographics and clinical characteristics of the study subjects

Variables	Total enrollment	Patients who underwent surgery
Total no.	904	388
Type of initial study site, *n* (%)		
Community	478 (52.9)	260 (67.0)
Referral center	426 (47.1)	128 (33.0)
Method of repeat biopsy, *n* (%)		
Fine‐needle aspiration	786 (86.9)	325 (83.8)
Core needle biopsy	152 (16.8)	78 (20.1)
Age of subjects, year		
Mean (SD)	48.1 (11.8)	46.7 (11.4)
Range	17–86	17–79
Sex, *n* (%)		
Male	192 (21.2)	76 (19.6)
Female	712 (78.8)	312 (80.4)
Nodule size on ultrasound		
Mean (SD), cm	1.2 (0.9)	1.2 (1.0)
Range, cm	0.2–9.4	0.2–5.9
Size group, *n* (%)		
<1.0 cm	438 (48.5)	204 (52.6)
1.0–1.9 cm	341 (37.7)	134 (34.5)
2.0–3.9 cm	104 (11.5)	38 (9.8)
≥ 4.0 cm	21 (2.3)	12 (3.1)

**Figure 1:**
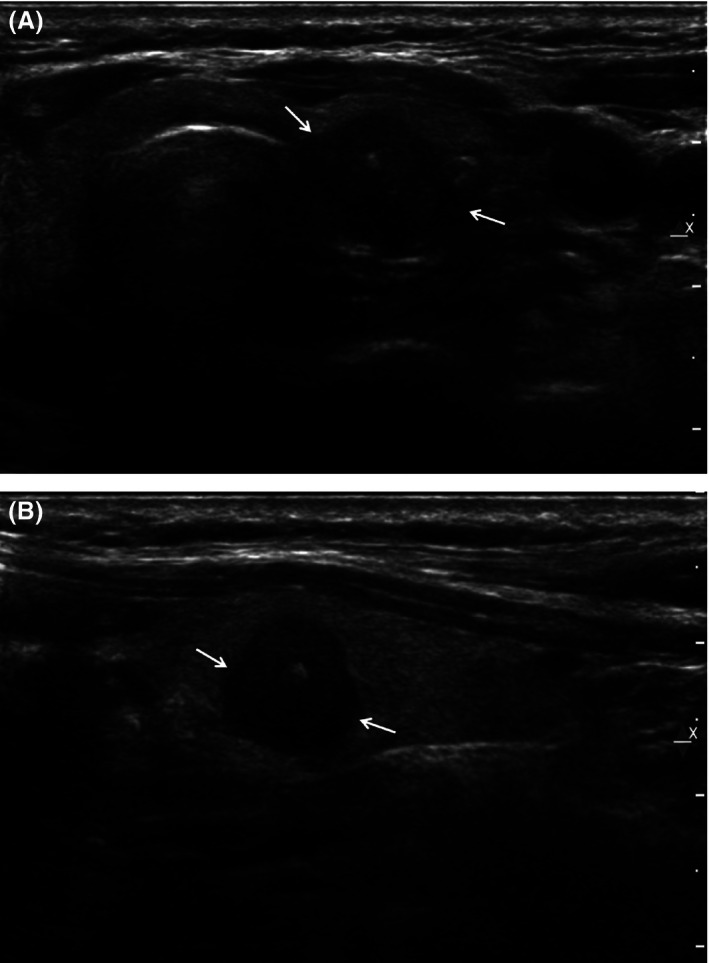
A 55‐year‐old‐female had a 1.0 cm sized irregular, marked hypoechoic nodule (arrows) with internal calcification in the left thyroid gland, which was suspicious for malignancy seen on US and atypia of undetermined significance (AUS) at the initial cytopathologic report (A. transverse image, B. longitudinal image). The result of repeat fine needle aspiration biopsy was still AUS and not conclusive. The *BRAF*^V^ ^600E^ mutation was detected in the aspiration specimen. Surgery confirmed papillary carcinoma.

Table 2 shows the distribution of repeat biopsy results according to the BSRTC categories. Repeat biopsy reclassified 79.0% of AUS/FLUS cases into a category other than AUS/FLUS or nondiagnostic (298 benign, 65 FN/SFN, 111 SMC, and 240 malignant cases). Of the 388 cases whose final histopathologic samples were available, 338 (87.1%) were diagnosed as cancer. Of the 351 cases with a category of SMC or above (SMC+) on repeat biopsy, 277 (78.9%) were cancer. The malignancy rate was 11.0% (61 cases) in the 553 cases with <SMC categories (Fig. 1) (*P *<* *0.05).

**Table 2:** Results of repeat biopsy and malignancy rates in each cytopathologic category

Repeat biopsy test results	No. of patients			Malignancy rate based on, %	
	Total enrollment	Patients who underwent surgery			
		Excised	Malignant	Total enrollment	Excision
Total	904 (100.0%)	388	338	37.4	87.1
Nondiagnostic	34 (3.8%)	6	6	17.6	100.0
Benign	298 (33.0%)	22	11	3.7	50.0
AUS/FLUS	156 (17.3%)	41	32	20.5	78.0
FN/SFN	65 (7.2%)	39	12	18.5	30.8
SMC	111 (12.3%)	85	82	73.9	96.5
Malignant	240 (26.5%)	195	195	81.3	100.0

AUS/FLUS, atypia (or follicular lesion) of undetermined significance; FN/SFN, follicular neoplasm/suspicious for follicular neoplasm; SMC, suspicious for malignant cell.

Table 3 compares the repeat biopsy, US, and *BRAF* ^V600E^ test results specifically for 388 cases with final pathology according to histopathologic subtype. A wide variety of malignant subtypes were correctly classified as SMC+, US‐suspicious, and *BRAF* ^V600E^‐positive according to each classifier. Of note, the US findings and *BRAF* ^V600E^ test did not agree in more than half of the cases with follicular variant papillary carcinoma or follicular/Hürthle cell carcinoma. Among 50 cases with benign histopathologic results, three and nine cases were falsely classified as SMC+ on repeat biopsy and US‐suspicious, respectively. Of 17 benign cases with available preoperative *BRAF* ^V600E^ results, one false positive was found. The final histology of this case was chronic lymphocytic thyroiditis, and its AS‐PCR test for *BRAF* ^V600E^ mutation was negative, but the *BRAF* ^V600E^ mutation was detected by highly sensitive MEMO sequencing of *BRAF* exon 15.

**Table 3:** Results of ultrasound, *BRAF* ^V600E^ mutation, and repeat biopsy according to histopathologic subtype

Histologic subtype	No. of patients (%)	Repeat biopsy	Ultrasound result	*BRAF* ^V600E^ result
		No. SMC+/no. <SMC	No. suspicious/no. nonsuspicious	No. positive/no. negative
Malignant				
Total	338 (100.0)			
Papillary carcinoma	272 (80.5)	240/32	218/54	98/26
Papillary carcinoma, follicular variant	55 (16.3)	33/22	20/35	8/22
Follicular carcinoma, minimally invasive	4 (1.2)	0/4	0/4	0/2
Hürthle cell carcinoma, minimally invasive	1 (0.3)	0/1	0/1	0/0
Medullary carcinoma	2 (0.6)	1/1	1/1	0/0
Poorly differentiated or anaplastic carcinoma	4 (1.2)	3/1	2/2	1/0
Benign				
Total	50 (100.0)			
Benign follicular nodule	23 (46.0)	2/21	5/18	0/7
Follicular adenoma	20 (40.0)	0/20	3/17	0/8
Hürthle cell adenoma	4 (8.0)	0/4	0/4	0/0
Chronic lymphocytic thyroiditis	3 (6.0)	1/2	1/2	1/1

SMC+, suspicious for malignant cell or above.

**Figure 2:**
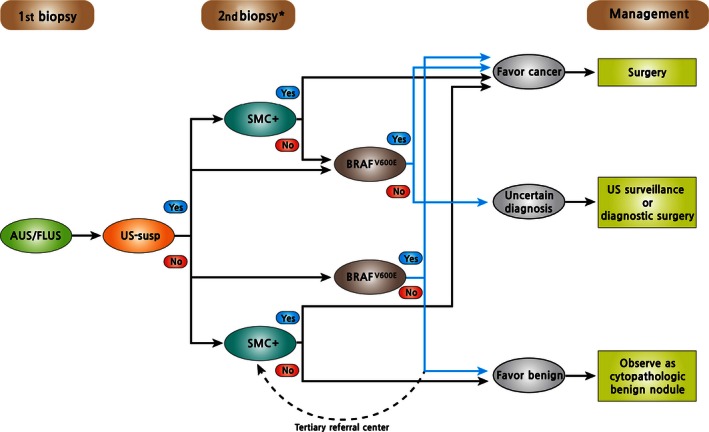
Suggested paradigm using multiple diagnostic tests to guide decision making in patients with AUS/FLUS on thyroid cytopathology. *For cases with follicular neoplasm/suspicious for follicular neoplasm on repeat biopsy, diagnostic surgery is the preferred option or consider other molecular testings beyond the *BRAF*^V^ ^600E^ mutation. AUS/FLUS, atypia (or follicular lesion) of undetermined significance; SMC+, suspicious for malignant cell or above; US‐susp, suspicious findings in ultrasound.

The sensitivity, specificity, positive and negative LRs, and estimated PPV and NPV of individual tests or as a set are presented in Table 4. The *BRAF* ^V600E^ and SMC+ on repeat biopsy had high positive LRs as a single test (11.6 and 13.7, respectively). The US features alone did not reliably classify either malignant or benign cases, as previously reported 16, 27. In particular, PPV of US‐suspicious was only 31% in the community center setting. Intersections of US‐suspicious and SMC+ revealed the highest positive LR (15.5) in this study but lower than that of the 7‐gene mutation panel 28. Unions of tests were generally useful for excluding malignancy surpassing the reported performance of gene expression classifier 29. The expected NPV for unions of tests exceeded 90% even in the tertiary referral center setting. Based on these data, a flow chart of a suggested paradigm for evaluation and management of AUS/FLUS lesions is presented in Figure 2.

**Table 4:** Performance of the diagnostic tests and predicted outcomes according to the prevalence of cancer in the AUS/FLUS category

Measurements	US‐susp	*BRAF* ^V600E^	SMC+	Intersections of tests			Unions of tests			The existing molecular panels	
				US‐susp and *BRAF* ^V600E^	US‐susp and SMC+	US‐susp and *BRAF* ^V600E^ and SMC+	US‐susp or *BRAF* ^V600E^	US‐susp or SMC+	US‐susp or *BRAF* ^V600E^ or SMC+	7‐gene mutation panel^a^	Gene expression classifier^b^
No. of participants	388	174	388	174	388	174	174	388	174	247	129
Sensitivity	71%	68%	82%	51%	62%	46%	87%	91%	94%	63%	90%
95% CI	(66–76)	(60–75)	(77–86)	(43–59)	(57–67)	(38–54)	(80–92)	(88–94)	(89–97)	(45–79)	(74–98)
Specificity	82%	94%	94%	94%	96%	94%	88%	80%	88%	99%	53%
95% CI	(69–91)	(71–100)	(83–99)	(71–100)	(86–100)	(71–100)	(64–99)	(66–90)	(64–99)	(96–100)	(43–63)
Positive LR	4.0	11.6	13.7	8.7	15.5	7.8	7.4	4.6	8.0	44.4	1.9
95% CI	(2.2–7.2)	(1.7–77.8)	(4.6–41.0)	(1.3–58.4)	(4.0–60.6)	(1.2–52.6)	(2.0–27.1)	(2.6–7.9)	(2.2–29.5)	(14.0–140.6)	(1.5–2.5)
Negative LR	0.35	0.34	0.19	0.52	0.39	0.58	0.15	0.11	0.06	0.38	0.18
95% CI	(0.28–0.43)	(0.26–0.44)	(0.15–0.24)	(0.43–0.64)	(0.34–0.46)	(0.48–0.69)	(0.10–0.23)	(0.08–0.16)	(0.03–0.13)	(0.24–0.58)	(0.06–0.54)
Test predictive values based on the prevalence of cancer in the AUS/FLUS category											
*10% (community health center)*											
PPV	31%	56%	60%	49%	63%	46%	45%	34%	47%	83%	18%
95% CI	(21–41)	(33–77)	(46–73)	(25–73)	(46–78)	(23–72)	(28–63)	(25–43)	(30–65)	(59–96)	(9–29)
NPV	96%	96%	98%	95%	96%	94%	98%	99%	99%	96%	98%
95% CI	(93–98)	(92–99)	(96–99)	(90–98)	(93–98)	(89–97)	(95–100)	(97–100)	(96–100)	(93–98)	(91–100)
*25% (secondary to tertiary referral center)*											
PPV	57%	79%	82%	74%	84%	72%	71%	60%	73%	94%	39%
95% CI	(48–66)	(63–91)	(73–89)	(55–88)	(73–91)	(52–87)	(57–83)	(52–68)	(59–84)	(82–99)	(28–51)
NPV	90%	90%	94%	85%	88%	84%	95%	96%	98%	89%	94%
95% CI	(85–93)	(84–94)	(91–96)	(78–91)	(84–92)	(77–89)	(90–98)	(93–98)	(93–100)	(84–93)	(84–99)
*40% (tertiary referral center)*											
PPV	73%	89%	90%	85%	91%	84%	83%	75%	84%	97%	56%
95% CI	(65–79)	(77–96)	(84–94)	(71–94)	(84–96)	(68–94)	(72–91)	(68–81)	(74–92)	(89–100)	(45–67)
NPV	81%	82%	89%	74%	79%	72%	91%	93%	96%	80%	89%
95% CI	(75–86)	(74–88)	(84–92)	(66–81)	(74–84)	(64–80)	(83–96)	(89–96)	(90–99)	(73–85)	(76–96)

AUS/FLUS, atypia (or follicular lesion) of undetermined significance; US‐susp, suspicious findings in ultrasound; SMC+, suspicious for malignant cell or above; LR, likelihood ratio; PPV, positive predictive value; NPV, negative predictive value.

a We used the tabulated data reported in the original publication by Dr. Nikiforov and colleagues (Fig. 2) 28.

b We used the tabulated data reported in the original publication by Dr. Alexander and colleagues (Fig. 1) 29.

## Discussion

In this study, we addressed the question how best to integrate the multiple tests into a triage of a nodule with AUS/FLUS at an initial FNA. Overall, SMC+ on repeat biopsy or *BRAF* ^V600E^ mutation in cytopathologic specimen should be a trustworthy surrogate for cancer risk and therefore be considered for surgery (i.e., total thyroidectomy). Absence of suspicious findings in US combined with <SMC or negative *BRAF* ^V600E^ mutation produced over 90% NPV both in the primary care and referral center settings.

We intended to establish a simple triage scheme based on widely available information in the routine practice of the thyroid clinic. The scheme included US findings, the results of repeat biopsy or the *BRAF* ^V600E^ mutation in cytopathologic specimens. Components of this scheme are easily comprehensible and underscore the importance of this staple information in the management of thyroid nodules. Although there is a guideline for treatment decisions using sophisticated, still limited‐use molecular kits 24, this approach provides a level of certainty to the initial AUS/FLUS results for individual patients, and it may also decrease unnecessary surgery for benign cases or two‐stage completion surgery for cancer.

The classification using the BSRTC system has proven highly beneficial, allowing better communication with standardized terminology among practitioners from various institutions or professions involved in the management of thyroid nodules 1, 30. However, with regard to the AUS/FLUS, it represents a heterogeneous diagnostic category for classifying thyroid FNA, and its use and follow‐up vary widely among institutions 31. The use of the AUS/FLUS diagnosis is suggested to be kept to a minimum (~7% or fewer of all thyroid FNA), similar to that of atypical squamous cells of undetermined significance (ASCUS) in cervical Pap smear 5, 32. However, forced application of this threshold should be another source of conflict between the diagnosis by cytopathologists in primary care and referral centers managing the same patient.

Aside from the inherent limitation of interobserver heterogeneity, the risk of malignancy of an AUS/FLUS nodule is difficult to ascertain because only a minority of this category had surgical follow‐up, and surgical criteria and management options differ among medical centers. In this study, the overall malignancy rate of the AUS/FLUS category calculated among all enrolled cases was 37.4% and that of resected cases was 87.1%. The high malignancy rate of the resected suggested that the selection was likely directed toward cases with clinically more aggressive diseases and this might limit the generalizability of the study results. As effort has been made to reduce unnecessary surgery for benign cases, the latter rate was undoubtedly an overestimate of the risk of all AUS/FLUS interpretations. Ideally, the risk of malignancy in the AUS/FLUS category should be independently defined at each institution to estimate the risk and to triage patients to either surgery or clinical follow‐up using the multimodal diagnostic techniques presented here. Assuming that clinically suspicious nodules, including a few lethal cancers, were more likely to undergo surgery early, the malignancy rate should be calculated based on total FNA cases, particularly for the cases with available cytopathologic or sonographic follow‐up data.

Clinicians may use an individual test item or available sets to triage an individual patient into categories that are more likely to be benign or more likely to be cancer. Clinical decision making based on the results of multiple inter‐related classifiers becomes easier by the intersection or union approach, which reduces the number of test variables to a dichotomous result 33. In this study, the union approaches using multiple test results produced robustly low negative LRs, allowing NPVs of more than 90% even in the tertiary referral setting. The positive LRs of SMC+ or *BRAF* ^V600E^ mutation were high enough to be used as a single test to triage patients to surgery and did not warrant the intersection approach, although SMC+ with US‐suspicious produced the highest positive LR. The absolute post‐test probability can be calculated with the information of pretest probability (malignancy rate in a nodule with AUS/FLUS in each institution) by using online calculators, Bayes' nomogram 34 or simply referring to Table 4.

It is important to note that this study has several limitations. First, the major caveat for the use of single gene mutation was its low sensitivity (68%) to detect malignant cases and negative LR of *BRAF* ^V600E^ mutation was only 0.34 that precludes its use as a sole test to exclude malignancy (NPV of 82% in tertiary referral center). Second, as in this study, follicular carcinoma or follicular variant of papillary carcinoma often shows indeterminate US findings and is negative for the *BRAF* ^V600E^ mutation. FNA also showed great difficulty in establishing a diagnosis for this entity 35, 36, 37. For cases with FN/SFN on repeat biopsy, the long‐standing preference of diagnostic surgery should be recommended, regardless of triage results 10. In this study, among the 39 patients who underwent surgery because of FN/SFN results on repeat biopsy, 12 had cancer and one had poorly differentiated carcinoma. A recently developed next‐generation sequencing panel of genetic markers may be considered to supplement malignancy risk assessment of FN 38. Third, because the majority of enrolled patients did not undergo surgery during the relatively short follow‐up period, a certain portion of malignant cases had not yet received surgery, so the risk of malignancy would be underestimated in this population. However, these cases are more likely to be characteristic of asymptomatic thyroid cancer in which aggressive management would not be appropriate. Fourth, although our results are useful for estimating the malignancy risk given particular diagnostic tests, they do not provide any information about the efficacy of the triage scheme in reducing thyroid cancer morbidity.

In conclusion, clinical decisions should be individualized with integrated interpretation of multiple diagnostic test results for assessing the likelihood of cancer. The simple triage scheme based on US findings, repeat biopsy or *BRAF* ^V600E^ mutation in biopsied samples can be used to identify a subpopulation of patients with low or high likelihood of cancer in a population of patients with AUS/FLUS at initial FNA in both primary care and referral center settings.

## Conflict of Interest

None declared.
